# “*We Sometimes Hold on to Ours*” – Professionals’ Views on Factors that both Delay and Facilitate Transition to Adult Care

**DOI:** 10.3389/fped.2016.00125

**Published:** 2016-11-24

**Authors:** Susie Aldiss, Hilary Cass, Judith Ellis, Faith Gibson

**Affiliations:** ^1^Faculty of Health and Medical Sciences, School of Health Sciences, University of Surrey, Guildford, UK; ^2^Evelina London Children’s Hospital, St Thomas’ Hospital, London, UK; ^3^Royal College of Pediatrics and Child Health, London, UK; ^4^Centre for Outcomes and Experiences Research in Children’s Health, Illness, and Disability, Great Ormond Street Hospital for Children NHS Foundation Trust, London, UK

**Keywords:** transition to adult care, adolescent, young adult, health professionals, focus groups, long-term conditions

## Abstract

**Background:**

The transition from child to adult services is a crucial time in the health of young people who may potentially fall into a poorly managed “care gap.” Health service provision, which fails to meet the needs of young people and families at this time of significant change, may result in deterioration in health or disengagement with services, which can have negative long-term consequences. Developing transitional care packages has become a focus of activity in the United Kingdom and elsewhere. Indeed, policy documents have been trying to guide practice for many years, with some variable success. There is much work still to be done, particularly around how guidance and the sharing of best practice, when combined can result in a change in practice.

**Objective:**

This study aimed to explore the views of professionals involved in transitional care, the process of transition in their services, and the barriers and facilitators to transition.

**Methods:**

This was a qualitative study using focus group methodology. Four focus groups were carried out, attended by 36 health professionals across child and adult services. They had expertise in working with young people with various health conditions and disabilities. Transcripts were analyzed using qualitative content analysis.

**Results:**

Eight key factors that impact on transition emerged from the data. These included factors associated with the patient group (such as age, health condition, having complex needs) as well as factors associated with services (such as the availability of equivalent services within adult care and the links between child and adult team).

**Conclusion:**

It is imperative that health professionals consider the population they are working with when planning transitional care and take into account the factors which can lead to delayed transition, so that this can be avoided if possible. Numerous examples of initiatives to facilitate more timely transition were shared: these have been reflected in our “Benchmarks for Transition from Child to Adult Health Services.” We offer these benchmarks to inform and guide the practice of others and illustrate their potential for use in the context of the findings shared here.

## Introduction

Transition services aim to bridge the “gap” between child and adult services. Despite a wealth of policy guidance in the United Kingdom (UK), preparation for transition is, however, often described as sub-optimal. Current approaches to transition are often described within three categories (1):

1. An abrupt transfer to adult services.
2. Staying in the pediatric area longer than is appropriate.
3. Leaving medical supervision altogether, voluntarily or by default.

All three are associated with impact, short and long term, on young people with a chronic illness as the receivers of these services. Simple transfer may result in increasing anxiety for young people, this immediate change in a relationship with professionals, often one that is long standing, can leave them feeling isolated from their normal support mechanisms, and they may worry that the adult health-care team will not be able to meet their needs. So for some, remaining with a health-care team they know may be their preferred choice. There is, however, the potential for delayed development into adulthood, and although they may feel safe in an environment and with people they know, some of their needs may not be met if they stay with a pediatric team too long. Disrupted care, or care that no longer meets their needs, can lead to disengagement from services and may result in deterioration in health. Transition programs are needed to enhance personal growth, increase control and independence by promoting skills in communication, decision making, and self-management. There is, however, no “one-size fits all model of transition” (2). That approach may indeed be inappropriate, as it may not consider variation in the young people themselves, or their preferred style of engagement (3). Personalized planning for transition seems more appropriate, where young people’s preferences, combined with the knowledge health-care professionals have of their patient population, could lead to more effective and efficient engagement with adult care. Certainly, reflecting on a comment made by Allen and Gregory in 2009 (4) might help us, as professionals, when we are thinking about transitional care: “rather than asking how best to manage transition, we might ask how best to meet the needs of young people with (a long-term condition) at this stage of their life course.”

There is, however, a significant and ever growing evidence base for the need for transitional care, what is limited is the corresponding evidence for how that care should be delivered (5). This lack of evidence leaves health professionals looking inwards on their own service and focusing on a single disease population: resulting in small scale service evaluations and the gathering of patient and family experiences, in order to inform local initiatives [for example, see Ref. (6–8)]. These local, single site initiatives have a role in service delivery and should be encouraged if we are to establish a body of robust evidence where we might eventually reach agreement internationally on the key elements of transitional care (9). But how can we learn from such initiatives, what is the model for sharing best practice, other than from published studies? Although there is awareness among professionals that transition needs to be improved in order to enhance young peoples, parents, and carers experiences, it can be difficult for health professionals to know how to begin to make changes (10).

The benchmark model is one way for an organization to learn about its own practices, discover the practices of others, and make changes that will enable the organization to reach its goals: not intended to be only a general measurement of one organization (or part of an organization) against another, but it also includes the study and transfer of exemplary practice (11). Benchmarking originates from within industry. The basic principle of benchmarking is that a point for comparison is identified (a benchmark) against which all can compare (12). At the local level, benchmarking also encompasses: regularly comparing indicators; identifying differences in outcomes through inter-organizational visits; seeking out new approaches in order to make improvements that will have the greatest impact on outcomes; and monitoring indicators (13): supporting the attainment of patient-focused outcomes (14). Benchmarks can also be used at the strategic level, to better describe those areas where policy efforts should be concentrated to improve health-care system performance, and help to identify “gaps” where more research may be required.

Our study was concerned with the development of a clinical practice-benchmark “tool”; a “tool” that would combine evidence from research and policy, informing the points of comparison that would be used as the benchmark to facilitate transitional care (15). We sought the opinion of three discrete groups of experts in the development of the benchmarks: (a) group of health professionals involved in either researching transitional care or influencing policy and practice; (b) health-care professionals involved in the delivery of transition programs; and (c) young people aged 13–21 years with a long-term health condition and their parents. We focus here upon primary data collection with health professionals from group b, undertaken to explore the process of transition in the services they worked within and the barriers and facilitators to transition. Knowledge of services and the process of change within the UK National Health Service (NHS) mean that as a group of researchers we were aware of some transition initiatives, but we also know that local initiatives are not always shared nationally: we sought to learn more about these from group b.

The study aims were therefore:

1. To explore the views of health professionals who are involved in transitioning young people to adult health care.
2. To explore the barriers and facilitators to transition.

In this paper, we present findings that resulted from data collection with health professionals from group b. In the discussion, we draw upon the indicators of best practice from the benchmarks for transition (www.transitionstudy.co.uk) as evidence for the role and utility of benchmarks to support transitional care.

## Materials and Methods

### Setting and Participants

This study spanned four NHS Hospital Trusts in London, UK. Three of the hospital trusts provided general hospital services for children and adults living locally as well as specialist clinical services for patients from across London, the south east of England, and further afield. One hospital trust was a children’s hospital providing mainly specialist services to children from across the UK. The sites were all local to the research team, as it was anticipated that this would aid engagement and recruitment to the study. Health professionals working with young people at each site were invited to participate in a focus group *via* emails sent out by the local Principal Investigator at their site.

### Data Collection

With the intent to explore in-depth and wide-ranged experiences and perceptions, focus groups were used. They were our chosen method of data collection, used as a method in their own right, selected as shown to elicit rich information and insight through the dynamics of the interaction between participants (16). Each group was undertaken in the hospital setting and lasted 1–2 h. This included time for lunch/refreshments. The groups were moderated by two members of the research team (Susie Aldiss and Faith Gibson), using well-documented techniques of focus group methodology (16, 17). The focus group discussion used questions/prompts developed from the literature (Box 1) focusing upon the transition process within the service the professionals worked within and the barriers/facilitators to transition. This discussion was audio-recorded with permission. A summary, based on observational notes and a debriefing session, was undertaken by the moderator and the assistant moderator immediately after each interview. The interviews were transcribed verbatim in their entirety.

Box 1 **Focus group questions and prompts.**

1. When you think about adolescent transition, how would you describe where we are currently within your own service?
  - Are you happy with how transition services are developing?
  - Can you describe any enablers to support transition?
2. Does your service have a clear procedure to prepare young people for the transition to adult services?
  - Use of a readiness assessment questionnaire?
  - Use of documentation?
3. Do you have a transition program in place within your specialist area?
  - Written policy/pathway?
  - Are medical, psychosocial, and educational/vocational issues addressed?
4. For those of you that do not have a program in place, what preparation may be needed for the young person and their family?
5. Which professionals, even if not physically present in clinic, are actively involved in the provision of transitional care within your service?
6. Do you have specific transition clinics?
  - Involvement of multidisciplinary team?
  - Are adult services included?
7. Is there a team member who has a specific transitional care (or transition coordinating) role?
  - If yes, is this defined in their job description and job plan?
8. Do you feel you have enough time within your role to facilitate in transition within your service?
9. Can you describe any barriers you have faced when trying to facilitate transition?
10. If you had a successful transition program in place how would we know?

### Ethical Considerations

University Research Ethics Committee approved this phase of our study.

### Data Analysis

Qualitative content analysis was used (18): as it retains closeness to data from the focus groups and enables categories, which represents participants’ perceptions, to emerge in a systematic way. Analysis was undertaken by two members of the research team (Susie Aldiss and Faith Gibson). The transcripts were read many times allowing for familiarization with the focus group data, getting a general sense of the whole. It was clear that there were a number of factors discussed in each group that impacted on transition and two content areas were identified by the *a priori* study aims:

(1) What factors make transition more challenging and often result in delayed transition?
(2) What factors make transition easier and smoother?

Coding and categorization were then carried out inductively over several stages. First, the text within each content area was divided into meaning units, each comprising several words, sentences, or paragraphs containing aspects related to each other through their content and context: in order to explore our two content areas. Relevant comments were highlighted within the text and annotations were written in the margins. The highlighted comments (or summaries of the comments) were grouped into meaning units and transferred into a summary table. Second, taking the context into consideration, these meaning units were condensed and each was labeled with a code. Third, the codes were compared for similarities and differences and sorted into sub-categories and categories: thus, factors that both hinder and help transition could be explored within and across the focus groups.

### Findings

Four focus groups took place between August 2013 and June 2014. Thirty-six health professionals attended the groups, this included: 30 nurses, 5 doctors, and 1 allied health professional based within adult services, children’s services, or young people’s services. These health professionals had expertise in working with young people with many different health conditions and disabilities (specialties listed included rheumatology, diabetes, respiratory, hematology, neuro-disability, safeguarding, neurology, cardiac, renal, gastroenterology, HIV, cystic fibrosis, oncology, and allergy).

Eight key factors that impact on transition emerged from the data (Table 1). These factors are discussed in turn with quotes from the focus group participants to illustrate each factor. In this text, “transition” refers to the process of moving to adult care, preparation for which starts within child health services and continues in adult services. “Transfer” refers to the point at which the young person moves to adult health services and is discharged from child health services.

**Table 1:** **Factors that impact on transition to adult care**.

Key factor that impacts on transition	Sub-factors that make transition challenging	Sub-factors that facilitate a smoother, timelier transition
Young person’s age	The adult service has strict age criteria making transfer before this age not possible even if the young person is ready to move. Different services have different age criteria – if a young person moves to adult services this can create issues accessing other services, which still fall under pediatrics. 16- to 19-year olds fall between services generally.	There is an age restriction in children’s services meaning young people have to move on. There is an established process for transition with a clear start and end point.

Length of relationship between professionals and the young person/parents	Long-standing relationship – the professionals/team have known the young person all their life.	If a young person is diagnosed during adolescence sometimes it is appropriate for them to see the adult team straightaway.

Transfer of responsibility for health to the young person	The young person is not fully informed about their condition. The young person is not seen in clinic very often making preparation for transition difficult. The young person does not begin to take on responsibility for their own health and is reliant upon parent(s).	The pediatric team work gradually with the young person to prepare them for self-management. If a young person is diagnosed during adolescence, they take more responsibility for their own health.

Service provision	No equivalent adult service to transfer to. Issues with commissioning services. Transitioning to multiple teams – children’s service covers a large geographical area, the team do not know or good links with all teams young people are transferred to. Adult team do not cover all the young person’s needs – do not deal with issues such as school/college.	The adult service is perceived as “good” by the pediatric team. There are enthusiastic “key people” to work with in the adult team. The pediatric and adult team have a good relationship. There is a “young adult” service to transfer young people to.

The young person has complex needs	The young person requires support from multiple teams.	The young person does not have complex needs.

Pathway/guidelines	Lack of clear guidelines/pathway makes transition fragmented – different services within the same Trust undertake transition differently and at different times.	When a service has a well-established pathway/guidelines in place

The young person’s health condition	The young person has relapsed. The young person is near the end of treatment. The young person requires psychological support which is not as accessible within the adult service. Young people with neurological disabilities where there is a lack of provision for a parent/carer to stay with them as an inpatient.	The young person’s health condition is stable and relatively straightforward.

Involvement of the multidisciplinary team (MDT) in transition	Lack of involvement of MDT in the transition process.	MDT are involved in the transition process.

### Young Person’s Age

There was a lot of discussion in the focus groups around “age.” Although it was perceived as useful for services to have clear guidelines about patient age, some flexibility was welcomed as rigid guidelines made transition more challenging in some circumstances. If clear guidelines were not in place about when transition should occur, sometimes services would delay transfer for as long as possible, which could lead to a rushed process where the young person is not prepared,

At eighteen we have to almost force them out, because we have this dilemma that we cannot admit them should they become ill, because of the whole bed crisis in this place that much after sixteen. If you don’t prepare them before, you end up with this dilemma where they maybe sick and if they get admitted from clinic to the ward, they will get transferred very quickly to (name of hospital) and get put on an adult unit without any preparation. (Transcript [T]1)

Some adult services would not see young people until a certain age, which meant transfer was delayed for some young people who were ready to move on earlier,

We’ve also had a couple of scenarios where maybe some of your young people were actually ready to transition before they turned eighteen and wanted to be seen at the adult clinics but actually the adult setting couldn’t until they were eighteen. (T1)

Age presented a particular challenge for transitioning young people who access more than one service as often these services had different age criteria,

The majority would come (to the adult service) at the age of sixteen. If I then want to refer to palliative team for argument sake, it is a lot of battle, because they are not in the adult year, because in the community they are probably assessing them at the age of eighteen or nineteen. They are still technically under pediatrics. (T2)

This was seen as a particular issue for young people aged 16–19 years who seemed to fall between some services.

### Length of Relationship between Professionals and the Young Person/Parents

Many of the health professionals spoke about how it was more difficult to transition patients, “*when you have known some of them literally almost all their lives*” (T1). This long-standing relationship made young people and their parents reluctant to leave the service and services more likely to delay transition and “*hang on to them longer*” (T3).

I have eighteen year olds that won’t leave and I have-, we keep them on under a lot of pretexts of they need to wait for the University results, so come the summer, they turn eighteen in April, what is the harm of giving them another appointment? (T1)

Some health professionals felt it was parents who found transition more difficult and recognized that parents need to be supported through the process. Part of this issue was around “*trust*” and forming a relationship with the new team. Delaying transition was identified as exacerbating this issue as,

That has the knock on affect then, because the new adult team hasn’t had the time to build the relationship, and get to know them. So that’s where the problem is. (T3)

A few examples were given where young people still contacted the pediatric team, even once they have moved to adult services,

All of these things are in place, where actually there are still patients that want to get hold of me, and just the other week, there was a patient who emailed me first for a reference or something. I emailed her back saying, ‘I haven’t seen you for a year, and you should contact to see the nurse that you’re with, in the adult site.’ Then she still wanted to meet me for coffee, and sort of telling me things that, maybe, she hadn’t told her adult nurse. (T3)

### Transfer of Responsibility for Health to the Young Person

In order to successfully move on to adult services and an independent adult life, young people need to take responsibility for their own health. Health professionals working with young people who have HIV or who had cancer at a young age described some instances where a young person was not fully aware of their condition which made preparation for self-management difficult. There were also some young people who did not currently wish to be more independent from their parents, for example, their parents still managed their medication or when offered time alone with the doctor/nurse they declined and wanted their parent to stay,

I’ve worked with patients who are very happy for their parents to do that for them. You know, it’s anything with life, isn’t it, and actually their parents are much more bothered about it than they are lots of the time. Ours would happily come with their parents to all the clinics. (T3)

Young people were more likely to self-manage if they had a health condition diagnosed in adolescence, rather than when they were younger, as they had been encouraged to do this from diagnosis,

I think that’s another difference between a chronic illness that you’re born with, and getting cancer as a teenager, because we hope to empower them from diagnosis. So because they’re diagnosed in adolescence, they’re treated very much as an adolescent. Whereas yours is about, ‘Do you know what tablets you take,’ because, you know, their mums have always managed them. So I think it’s slightly different, that moving on. (T3)

Frequency of appointments impacted on preparation for self-management and transition, in some services young people attended clinic every few years, “*so that is quite difficult, because it doesn’t really give you very long to do all your transition stuff in*” (T1).

Other services has gradual preparation for all young people in place and professionals saw it as their responsibility to help the young person work toward independence and self-management,

I think it’s my responsibility that they’ve got to understand their illness because especially if they’ve got a transplant that’s not going to last forever, they might end up being back on dialysis and getting re-transplants. It’s a part of my job to make sure even if they were dialyzed for the first two years of age and then got a transplant and then they’re coming and seeing me every three months, they’ve got to understand what impact that might have for their long-term life as well as their long-term health. (T1)

### Service Provision

The commissioning of services created barriers around where young people could be transitioned to and whether patients were accessing a “local” or “specialist” regional service. Sometimes, an equivalent adult service did not exist to transfer young people to; therefore children’s services tried to delay discharge as long as possible.

Transition was much smoother when the pediatric team had a good relationship with the adult team. Teams needed to form links with enthusiastic “*key people*” interested in working with young adults who can “*fly the flag in their adult service*” (T1). This was only possible when the pediatric service transitioned patients to a limited number of adult teams, which were fairly local. For some specialist children’s services covering a large geographical area, sometimes the whole of England, it was not possible for them to form good links with all possible teams a young person may move on to.

Children come to us from all over England. So when they need to go to adult services, if they don’t want to continue coming to London. Or, if they’re going to their university hospital, then transition suddenly gets a little bit more tricky, because you’re not just dealing with one adult team. You’re dealing with a hospital, potentially, at the other side of the country. So, you’ve got patients from all over the place, so it’s not just one, you’re doing it for all of them. So that’s when it falls down slightly, and it’s harder to have a smooth transition where you meet up with the other team, because it’s just not physically possible. (T3)

Even when young people have moved on to adult services, there were examples of when they still needed help from children’s services as the adult service did not cater for all their needs,

If they are still at school or college, the sort of thing I would often be doing is speaking to their schools or their colleges and explaining how their condition affects them, writing letters for the extra time in their A-levels and all that sort of thing. They don’t seem to have people that would do that so well in the adult services, so I am saying, ‘Yes, you are going to see the adult doctor now, but if you need this, just come back to me and I will do it for you,’ when officially they have been discharged from pediatrics. (T2)

The availability a young adult service to transfer young people to was viewed positively, as it was thought that these services catered more to a young person’s needs.

### The Young Person Has Complex Needs

There was some discussion around transition for young people with some conditions being more straightforward (such as diabetes). When a young person has complex needs, which necessitated the involvement of several different specialties, transition was complicated. Sometimes, the equivalent teams which were within the same hospital for children’s services could be in different hospitals for an adult.

I have currently been working with one of our mums, only because I think she just feels completely overwhelmed by the number of teams and with working with another colleague, we have broke it down between us and I think at that, I think we were trying to contact five different teams, so that is ten. We have all talked individually about our own transition plans for our patients, but that family could be doing that ten times over, with ten different teams and it is overwhelming. (T1)

Ideally, timing of transition and transfer needs to be coordinated between all teams the young person was seen by; however, this was difficult in practice.

They’re seen by about five different doctors in this hospital, and they’re still coming back for this, so they should come back. That’s always the excuse. (T3)

Psychology is only 18 if they’re still under a pediatric consultant…. So if they move at 16 to an adult consultant then child psychology can’t still see them. (T4)

### Pathway/Guidelines

Some health professionals worked within services that had a well-established process for transition, which was documented in written guidelines/a pathway; this made transition much smoother. However, there were still issues when a young person accessed more than one service as the pathways were not trust-wide and services used different documentation. There was discussion around transition being “*fragmented*” and nothing being “*standard*” (T4). Contributing to the issue was health professionals having different views about the ideal age for transfer.

If we had an age that everyone worked towards, and we all did the same, it might be a bit easier. (T3)

There was some discussion about how transition needed to be high on the agenda of senior health professionals in the trust, in order to drive forward change and implement more generic, trust-wide pathways.

### The Young Person’s Health Condition

There were certain time points in the young person’s illness that made it more difficult to move them on to adult services, resulting in transition being delayed, for example, when a young person had relapsed or was near the end of their treatment,

We always talk about transition, and of course it always happens when they relapse, or are terribly tired, and you think, ‘Oh my goodness, they’re twenty, and we haven’t handed them on.’ So, of course, you get this poor person who has relapsed, and have now got to change complete teams…. I think as a CNS it’s quite hard, if they’re just relapsing, to move them on, when you’ve had a relationship with them for a long time. (T3)

There are sometimes that, I think, we’re a bit reluctant to transition ours, but that’s, I suppose, that’s where cancer is different. Say, for example, if they’ve only got six months left until the end of their treatment, we’ll try and keep hold of them, because what’s the point of traumatizing them by transferring them. (T3)

The use of other services, which may not be available in adult services also impacted on timing of transfer,

We sometimes hold on to ours, as well. For instance, if they’re having a psychological problem, and they need continuous psychology, because that cuts off at eighteen. If they’ve already started, the psychology team will continue, until transition. So we don’t kind of cut it if we feel that they’ve still got a need there. (T3)

There was discussion around parents/carers not being able to stay when a young person is admitted to an adult ward. This was a particular issue for young people with severe neurological disabilities who relied upon their parents to care for them. This made services more likely to try to delay transfer as long as possible.

### Involvement of the Multidisciplinary Team in Transition

Transition in the services the health professionals worked within was mainly nurse led. There was frustration expressed that other members of their teams did not get involved in transition. This lack of involvement made transition more difficult particularly when information from another health professional group (such as dieticians) needed to be shared with the new adult team,

It’s important that you’ve got a whole multi-disciplined team and yet there’s one person leading it. We’re trying to make it holistic for the patient when it’s not holistic if it’s just the nurses doing it so they need real pushing. (T1)

Again, we’ve got the problem with other professional groups who are not really interested in transition. (T1)

## Discussion

Within and across the focus groups, health professionals expressed concerns that the care provided to young people should be developmentally appropriate (19). They felt young people should be given the opportunity to move to adult services and not remain too long in children’s services when this is no longer appropriate. Certainly, we know that late transition can lead to poor patient outcomes mainly due to the late exposure to the adult care settings and lack of independence (20, 21). Several key factors that either delay transition or facilitate a smoother, timelier transition were evident from our data. Many of these factors related to the young person – their diagnosis, length of illness, needs, and age. We would therefore argue that it is imperative that health professionals consider the population they are working with when planning transitional care for young people. Although much of the discussion in the focus groups was around barriers to transition and the factors that lead to young people having a delayed transition, many examples of initiatives to improve transition were also shared. It was evident that there had been little sharing of these strategies between teams, as health professionals working within the same hospital were unaware of what colleagues from different specialties had already implemented. As a support to change, we could see that benchmarking could have a clear role in helping health professionals to apply best practices (22). We offer here some reflections on our findings, drawing upon published work and then place our benchmarking “tool” in the context of these findings to illustrate its potential role to impact on structure and processes, and conclude with some suggestions for how benchmarks might also identify patient benefit.

In order to facilitate transition, there needed to be enthusiastic key people working within both the pediatric service young people were being transitioned from and the adult services they were moving to. In services where there was a good relationship between these teams, the process of transition was much smoother, and transition was less likely to be delayed. These were often services, which were local to each other, had worked together for a long time and had an active well-established process of preparation in place, supported by a written pathway. All the groups spoke about a lack of coherence across services within their trust, which made transition challenging even for services, which had their own established process in place. There was a call for trust-wide pathways and national guidance to aid a more coordinated transition experience when a young person was accessing more than one service. Since our study was completed, National Institute for Health and Care Excellence (NICE) guidance (23) on transition has been published, it is too soon, however, to determine whether this guidance will influence changes in practice when so many of the previous transition guidance documents have failed to do so (24).

The health professionals spoke about how hard it was to transition patients they had known all their life, they wanted to “hang on” to these patients for longer. They worried about whether “their” patients’ needs would be met in the adult service. This was a particular issue for those who have complex needs. It was viewed as important that the young person and family were given the opportunity to build a relationship and establish trust over a period of time with the adult team, prior to be being transferred. This was achieved through: joint clinics being held with members of both the adult and child teams, being accompanied by a member of the child team on the young person’s first visit to the adult clinic, having accompanied visits to the adult inpatient area and having the chance to come back to see the child team following the first appointment within adult services to check all was going well with the transition. A few services gave young people the chance to visit multiple adult services before deciding which they would like to transfer to. In particular, someone to take responsibility for a young person’s transition was deemed to be important, a “key worker” or coordinator, who would oversee the transition (25–27). However, this raised funding issues, where these roles might be based within children’s services and are therefore not picked up adequately when a young person transfers to adult care. As McDonagh and Gleeson suggest, the young person needs to be supported until they are “established” within the adult setting; “transition is only completed when young people are functioning competently within the adult service” [(28); p. 26].

As young people move into adulthood, responsibility for health needs to shift from the parent to the young person (29). Facilitating this transfer of responsibility for health to the young person was an issue faced by all the professionals. When young people were not fully aware of their diagnosis this was an impossible task. The health professionals described how they aimed to make this a gradual process, which started early. Some used checklists (30) to monitor progress in this area and give young people targets to work toward, such as making their own appointments. Having time alone with the doctor or nurse was seen as a vital step for young people to take to prepare them for the adult service and allow discussion of issues they may not wish to raise with their parents present (such as sexual health). Separating young people and parents was sometimes an issue, with young people being reluctant to be seen alone and parents being reluctant to leave them. One service had resolved this by offering a room and refreshments in clinic for parents to meet and speak together while the young people were seen in another consulting room. This enabled peer support and offered the young people time away from parents during the clinic. It was important to work with both the young person and parent together and recognize parents also need preparation for transition; they were sometimes viewed as finding the process more challenging than the young people. Previous research has reported that young people and parents do not always have the same views concerning transition (31), engagement with both parties is key to successful transition.

One aspect of preparing young people to take responsibility for their own health is the provision of information about their condition. Patient held records or health passports with a personalized health history were one way in which this information was provided (32). In some services, this was available through an app, rather than being paper based. There was some discussion about paper-based documents not always being successful, as they can be lost and sometimes the young person being reluctant to complete their sections. One health professional described how she had worked with a charity to develop a transition pack to be used nationally with young people so that everyone in the UK with that health condition could receive standardized information. Placing young people “in charge,” was a strong message through all our data and e-health initiatives are being developed, maximizing this concept to aid transitional care (33).

A few services held transition events as part of preparing young people for moving to adult care and managing their health as an adult. These events were a way of providing young people who did not have regular clinic appointments with information about transition. At these events more “adult” lifestyle topics around health and the impact of the health condition were discussed, such as use of alcohol, sexual health, driving and careers. Charities were often involved, to fund the event or be present to provide information. One issue raised in the focus groups was the lack of involvement of the multidisciplinary team in transition. High satisfaction has been linked to transitional care that is holistic (34). Transition events were a way to encourage involvement of the multidisciplinary team, team members were often encouraged to attend and provide input, where they would not usually be present in clinics. Some events also featured a “graduation ceremony” to celebrate the transition to adult care as a positive move.

**Figure 1:**
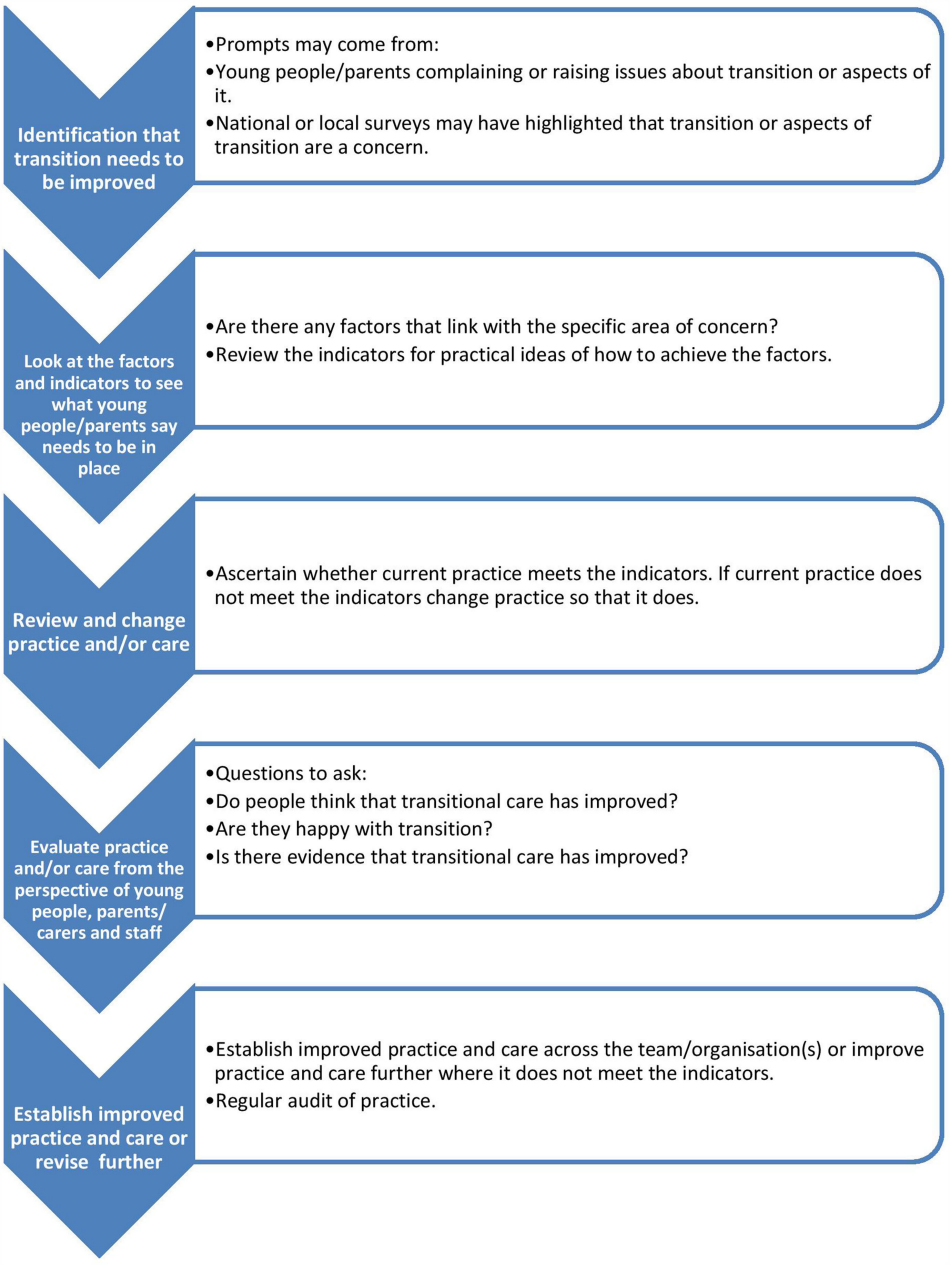
**Using the Benchmarks for Transition from Child to Adult Health Services**. ^1^Adapted from: Department of Health. How to Use Essence of Care (2010) The Stationery Office https://www.gov.uk/government/uploads/system/uploads/attachment_data/file/216690/dh_119970.pdf. Contains public sector information licensed under the Open Government Licence v2.0. http://www.nationalarchives.gov.uk/doc/open-government-licence/version/2/.

We were privileged to hear about local creative initiatives that were focused on enabling the young person and their parents to develop skills and seek support when needed, in order to self-manage, get on with their life in the knowledge that appropriate hospital services were available to them. But, we also heard accounts about the barriers, from health professionals themselves as well as those created by service configurations that made individualized, efficient, and effective transition difficult. In terms of services, sustainable, variable, and equitable were concerns raised through this period of data collection. There was, however, evidence of good examples of best practice to be shared beyond these four sites. The “Benchmarks for Transition from Child to Adult Health Services” (Table 2), produced as the output from our main study, developed with health professionals, young people and parents, provides services such as those we worked with best practice statements to learn with others, to share with others, to develop innovative and new practices to meet the needs of their patients. The factors in the benchmark, although yet to be systematically evaluated, when used alongside methods inherent in quality improvement training, should allow clinical teams to both question and revise their practices (Figure 1).

**Table 2:** **Factors from the Benchmarks for Transition from Child to Adult Health Services**.

Factor	Best practice
Factor 1: Moving to manage a health condition as an adult	Young people are offered advice and information in a clear and concise manner about how to manage their health condition as an adult
Factor 2: Support for gradual transition	The young person as they progress through the transition process is gradually prepared and provided with personally understandable information and support
Factor 3: Coordinated child and adult teams	The young person is supported through a smooth transition by knowledgeable and coordinated child and adult teams
Factor 4: Services “young people friendly”	Young people are provided with care and in an environment that recognizes and respects that they are a “young person,” not a child or adult
Factor 5: Written documentation	Concise, consistent and clear written document containing all relevant information about the young person’s transition is provided to the teams involved in the transition process
Factor 6: Parents	Parents are included in the transition process gradually transferring responsibility for health to the young person
Factor 7: Assessment of “readiness”	The young person’s readiness for transition to adult care is assessed
Factor 8: Involvement of the GP	The young person’s GP is informed of the plan for transition and is able to liaise with other relevant teams to facilitate services requested/needed by the young person

The benchmarks will enable practitioners to share common difficulties and to offer each other practical support and encouragement, in this case when developing and implementing transitional care. They will also support health professionals in effectively meeting patients’ needs and supports practitioners in a continuous cycle of comparison and sharing that is aimed at ensuring young people and their families receive evidence based care, duplication of effort is avoided and the efficient use of resources are encouraged. So, for example, written documentation (Factor 5) and patient information aimed at young people to get them ready for transition could be trust (hospital wide) as opposed to disease specific. Templates for patient information, patient passports, algorithms that assess transition readiness (Factor 7) and generic guidance for self-management (Factor 1) can all be developed hospital wide, benchmarking nationally with other organizations might reveal “better” templates, or indeed some creative solutions to local challenges. Many of our participants reflected on the role of parents. We know that the changing role to support the child’s independence by taking on adult roles and responsibilities is not always easy for parents (35), for young people or health professionals (36): where the “parent as partner” role has been described (37). Tensions known to occur, where a shift to the young person as the central decision-maker, we suggest might be more easily recognized and enabled through use of the benchmarks (Factor 6). Addressing the transitional care needs of parents as well as young people is essential to facilitate a “shared care” approach as an intermediary measure that supports transition of both parents and the young person (38). Being gradually prepared (Factor 2) to move into adult roles with a developmentally appropriate approach, through collaboration between pediatric and adult teams (Factor 3), will require some approach to “overlap” to help connect the two systems and bridge the perceived “care gap” (39). Primary care involvement was mentioned by our participants: in the UK this would be the role of the general practitioner (GP), and although an initial contentious inclusion in our benchmarks (15), there was a final recognition of the importance of this role (Factor 8). Certainly, NICE Guidance (23) confirms the importance of primary care, and although there is limited empirical evidence to guide primary care interventions (40), we know that system level changes are needed to improve transitional care: hopefully, the benchmarks will go some way to support this. Reinventing the wheel is thus reduced through use of the benchmarks, and health professionals can benefit from others progress. Patients and families also benefit, where more consistent approach to practices becomes possible, and even those with a rare disease can gain from what has been learned with other patient populations.

Health professionals in this part of our study called for clearer guidance and pathways. They were describing local solutions to local problems. But what they and their patient populations are facing could be shared more widely: certainly in the UK the recent NICE guidance is much needed (23) if we are to address the priorities set by the CQC (24) that:

1. Commissioners and providers must listen to, involve and learn from young people and their families and understand what they want from their care.
2. Existing good practice guides must be followed to ensure young people are properly supported through transition.
3. GPs should be more involved at an earlier stage, in planning for transition.
4. Adolescence/young adulthood should be recognized across the health service as an important developmental phase.

We offer to our international audience the benchmarks and would encourage you to use, adapt, revise, and share to inform your own practice: using the benchmarks within the multi-professional team and with patients and families to inform both structure and processes to shape transitional care. Only through use will we be able to start to describe patient benefit, often not assessed or measured in benchmarking initiatives (41), but essential if we are to capture experiences of care to inform change that is centered on the receivers of our care. We know from our initial pilot that these benchmarks need adapting to specific populations, such as those with complex needs/disabilities (42), and mental health needs (43), and we would encourage readers to do that to ensure that care philosophies, service delivery, and transitions are a good fit with the population, particularly for those who require services from multiple agencies (44).

### Strengths and Limitations of the Study

The findings reported here represent the views of a limited number of health professionals who self-selected to take part. The participants were mainly nurses who were interested in transition and were trying to implement or had implemented initiatives to improve transitional care within their services. Discussions were very similar across all focus groups, which suggest some consensus on the current challenges around this topic. The discussions also resonated with issues relating to transition previously reported in the literature (45). It is of course important to not only seek the views of professionals around transition but also include users – young people and parents. This work did include user perspectives, which are reported elsewhere [(15), www.transitionstudy.co.uk].

## Conclusion

In order to develop appropriate transitional care for young people, evidence of what works and what factors act as barriers and facilitators of local, national, and international initiatives are much needed. We offer in this paper, some reflections on practice from a small group of health professionals working in the UK, to reveal challenges that exist at both service and professional level: evidence that both structure and processes are needed to be in place to facilitate transitional care. Local initiatives were encouraging, we share those and some examples from others, as indicators for best practice in the benchmark document. We strongly recommend the use of the benchmarks, as they stand if they are a good fit for use, or adapted if required to reflect the specific needs of a population or an international perspective. We have more in common than different when we reflect on our patient populations, and although service configurations differ hugely, if we focus on what young people need, the benchmarks can have a significant role in shaping transitional care. Better-tailored approaches to transition is key if we are to bridge the “care-gap” between child and adult services described in the literature: interventions that teach life skills and self-management need to be evaluated; strategies that improve collaboration between services and enhance a shared understanding of approaches to care must be described and made known; different models of transitional care need to be examined; and finally, we need to know more from the populations we care for about their experiences.

## Author Contributions

SA set up the study in the sites. SA, FG carried out data collection. SA, FG carried out data analysis. HC, JE were involved in interpretation of findings. SA, FG prepared the manuscript. All authors reviewed the manuscript and approved the final version.

## Conflict of Interest Statement

The authors declare that the research was conducted in the absence of any commercial or financial relationships that could be construed as a potential conflict of interest.

## Abbreviations

MDT, multidisciplinary team; NHS, National Health Service; NICE, National Institute for Health and Care Excellence; T, Transcript; UK, United Kingdom.
